# 

**Published:** 1892-09

**Authors:** 


					CONTENTS.
PAGE I
Forceps Delivery during the last
Fifty Years.
By Joseph Griffiths
Swayne, M.D. Lond.
153
A Case of Large Ovarian Tumour in
a Young Girl.
By J. Macpherson
Lawrie, M.D., C.M. Glasg.
186
A Case of Multiple Symmetrical
Joint Lesions.
By Charles A.
Morton, F.R.C.S. Eng.
189
Progress of the Medical Sciences:
Medicine.
By R. Shingleton
Smith, M.D..
195
Surgery.
By W. J. Penny.
201
Ophthalmology.
By F. R. Cross
208
Reviews of Books
211
Notes on Preparations for the Sick
222
The Library of the Bristol Medico-
Chirurgical Society   223
Scraps
227

				

